# The current three-year postgraduate program in urology is insufficient to train urologists: Against the motion

**DOI:** 10.4103/0970-1591.42615

**Published:** 2008

**Authors:** Pankaj Wadhwa

**Affiliations:** Department of Urology, St. Stephen's Hospital, Delhi-110 054, India

“Education is a kind of continuing dialogue, and a dialogue assumes different points of view.”
- **Robert M. Hutchins**

Surgical practice, especially urology, has been significantly affected by rapid innovation in medical and surgical therapies. This has consequently impacted the delivery of urological care which is further modulated by available resources, patient demographics and expectations. There is thus a constant challenge to the urological education programs that are continually striving to provide comprehensive training in the ever-expanding technologies and techniques. The objectives of the residency training should be congruent with these changes. Hence the goal of this debate, in my opinion, should be aimed to examine the utility of the current urological training pattern in its existent timeframe as they pertain to today's urological realities.

## UROLOGY IN INDIA: EXISTING INFRASTRUCTURE AND PROJECTED NEEDS

As of February 2008, there are 1749 trained and registered urologists in India,[1] serving a population of 1.12 billion,[2] i.e. a ratio of 1: 646,007. To put this figure into clearer perspective, in 2002, the British Association of Urological Surgeons (BAUS) estimated a required consultant ratio to population ratio of 1:80,000 to be able to provide a reasonable standard of urological service in the UK for the year 2002.[3] Needless to say we are grossly inadequate in numbers to serve our country's urological needs. This prompts the question, ‘What are our urological needs as a country?’ Collated data showing what ails us collectively in this regard was unavailable, however, data regarding patients treated over a one-year period, at three large tertiary care centers in Delhi, catering to differing socioeconomic strata was collected from records and compared to estimate the urological case load encountered[4–6] [Table 1]. The limitation of the data stems from the skewed population representation, as it has geographical limitations of the population treated, as also the possible variability of patient referral patterns to these hospitals. The three institutions were deliberately chosen to reduce the skew in the referral patterns—a premier government-aided teaching hospital with an MCh course in urology, a semi-private teaching hospital without any teaching course in urology, and a premier private hospital with a DNB program in urology.

**Table 1:** Comparative data of operative workload from the urology departments of AIIMS, St. Stephen's Hospital and Sir Ganga Ram Hospital

Procedure	AIIMS (n=6826)	St Stephen’s Hospital (n=742)	SGRH (n=1875)

	Percentages of all procedures		
Minor Surgery	77.3	29.39	37
Major surgery	22.7	70.1	67
Subgroup of major surgery Open surgery	23.3	11.4	10.8
Lower tract endourology (TURP, TURBT, VIU, Cystolithotripsy, etc)	47	44	47.7
PCNL (including upper tract endourology)	10.4	19.6	11.2
URS	5.8	9.4	12.5
Laparoscopic procedures	3.1	3	4.8
Robotic procedures	8.4	nil	Nil
Microsurgery	2	1.5	Nil
AV fistula	nil	10	Nil
Renal Transplant	nil	1.1	13

Despite the obvious difference in total case loads consequent to department size (number of consultants, bed allocation and number of OT per week available) the striking observation was the similar proportions of type of surgeries performed. The bulk of major surgery involved lower tract endourology followed by endourological management of urolithiasis and open surgery. The percentage of uro-oncology, especially advanced stage disease was expectedly higher at the AIIMS.

A closer look at the data available from St. Stephen's Hospital, which I believe serves as a median reference of the three as it predominantly caters to the middle socioeconomic section of society, showed that urolithiasis accounted for 30.7% of the total operative case load, Benign prostatic hyperplasia (BPH) accounted for 16.3%, urethral strictures for 14.6%, oncology-6% (predominantly bladder tumor), andrology- 1.5%, female urology-1.1% and access for renal replacement therapy-10.3%.[5]

Data available from Sir Ganga Ram Hospital revealed that urolithiasis formed 34% of the operative case load, BPH 33.1%, followed by uro-oncology 22.4%, renal transplantation 13%, reconstructive urology 4.4%, andrology 3.3% and pediatric urology (reconstructive) 2.4%.[6]

A similar study conducted in a single-site NHS trust hospital in the UK over a three-year period (2000-2002) revealed that diagnostic lower-tract endoscopy comprised 63% of all interventions, simple inguino-scrotal surgery 6.2%, penile surgery 6.4%, more complex inguino-scrotal surgery 1.1% and lower-tract endoscopy and manipulation 11.4%.[7] Upper tract manipulation contributed 4.5% to overall operative activity whilst only 7.4% of cumulative surgery involved complex, laparoscopic or reconstructive procedures.

With regard to emergency procedures required of a urologist, treatment of urosepsis, hematuria, obstructive anuria, genitourinary trauma, urinary retention, testicular torsion, and priapism are the usual conditions which should fall in the purview of a trained urologist.

Although the current data are from too small a sample to conclude what the differential requirements for urologists might be and there is need to estimate similar data from a large multicentric study to give valid results, one can roughly estimate the need for a general urologist with at least good upper and lower tract endoscopic skills, especially pertaining to treatment of urolithiasis, BPH and bladder tumor.

At the same time let there be no ambiguity in the minds of the reader that I strongly endorse the need to develop super-subspecialization to enhance the development of the various subspecialties in urology which will, in turn, upgrade the standard of the trainee urologist.

## EXISTING TRAINING SCHEDULES

The question is “Is the current Post Graduate (PG) program training our residents for what they will be doing in their subsequent practices?”

In the current PG program, a postgraduate general surgeon (three-year course) is inducted into the urological program (three-year) wherein he undergoes graduated training usually in the format of serving at different ‘workstations’- outpatient evaluation, emergency services, ward management, minor OT procedures, urodynamic evaluation, shock wave lithotripsy, use of ultrasound and minor USG-guided interventions, diagnostic uroradiology and finally operative urology. Some of these stations are served concurrently. Additionally, teaching rounds, case discussions, symposia presentation, journal evaluation and work audit usually complete the academic input requirements. A clinically oriented research protocol further provides an avenue for focused in-depth study.

With prior general surgical training, preferably with some experience as a surgical senior resident, the urological trainee is usually comfortable with abdominal anatomy and handling bowel, performing simple inguino-scrotal surgeries, understands tissue handling in general and may have basic laparoscopic surgical skills. Urological training aims to provide acquisition of improved uro-diagnostic capability, better understanding of uro-genital pathophysiology and uro-pharmacology, endourological skill, basic microsurgical skills, improve pelvic, perineal and retroperitoneal surgical skill, exposure to newer technological advances, renal replacement therapy options and surgical treatment of localized urological malignancies.

Thus the current program is geared to impart general urological training requisite for treating most urological disease prevalent in our population.

But are we expecting to teach all our trainees to perform complex, high-end or technologically advanced procedures such as robotic surgery, complex reconstructive procedures (neo-bladder formation/extensive urethral reconstructions) or advanced reconstructive laparoscopic procedures?

A good training program will provide exposure to complex and rare procedures, despite the fact that many of them would not be performing such procedures in their clinical practice. Yet, the knowledge gained may prove to be of use in emergency procedures, or while counseling a patient with possible treatment options.

The need of the hour remains a broadly trained general urologist capable of dealing with a broad range of urologic disorders both medically and surgically. Comprehensive training of general urologists is vital to sustaining our competitive role as the specialty primarily responsible for diagnosing and treating all diseases of the genitourinary tract.

This perception is also shared by urological trainers in Europe and the US.[8,9] Analysis of the record of in-training assessments (RITAs) revealed that the current training in the UK encouraged trainees (89%) towards subspecialization (predominantly uro-oncology-42%) which may not be appropriate to the service demand in the UK.[8]

There are concerns that the rapid progress in the field of uro-diagnosis, therapeutic options and surgical advancements cannot be effectively taught to the urological trainees.

“The object of education is to prepare the young to educate themselves throughout their lives.” Robert M. Hutchins

It is true that it is increasingly difficult to train all the trainees everything in the various urological subspecialties (endourology, laparoscopy, reconstructive, uro-oncology, andrology, neuro-urology, renal transplantation, pediatric urology, robotics, urogynecology). However, they can attain exposure as a rotation through the various broad subspecialties, allowing them to choose in the future the subspecialty of their choice through fellowship programs.

### What ails the system?

“We can ignore or deny that problems exist, contending that our traditional systems of training and delivery of service may need tweaking, but nothing more, in which case we will be mere observers, and remedies will be imposed on us” Peter Altman

33rd Presidential Address (2004) at American Pediatric Surgical Association

The problem starts with variability in the quality of teaching institutions available, lack of a unified goal-oriented teaching program, absence of a user-level (in-training) assessment of the program, ill-defined techniques of assessing competency (especially surgical competency).

Increasing the timeframe of the program alone, instead of addressing these issues is unlikely to achieve the desired result and at the same time may deter talent from pursuing the specialty. The training program should be able to raise the bar of competency of all its trainees to a basic common level, yet allow the more talented or interested trainee to excel.

Additionally, medical educationists believe that apart from technical/ professional competency all physicians should possess six core competencies: patient care, medical knowledge, practice-based learning and improvement, interpersonal and communication skills, professionalism and systems-based practice. However, I believe these core competencies need to be introduced right at the MBBS level, upgraded during MD/MS courses and followed up in the specialty training program.

## COMPARISON TO OTHER SYSTEMS: THOUGHTS ELSEWHERE

Tumultuous thought and debates on urological training are raging all over the globe. While the UK is contemplating a two-tier system, one focused on the practice of office urology, and one which is surgically oriented,[9] there are others in the US who believe this may prove counterproductive.[10] Although there is considerable interest in reducing the existing urological training on the basis that a significant number of urologists in the US practice office-based urology, endoscopic and minor procedures,[11] others feel that any such change in the residency program in terms of length or content focusing on more limited training should be accompanied by simultaneous plans to ensure the need for training adequate urologists capable of more complex surgeries.[7,12]

## THE WAY AHEAD…

1. Reduction in the marked variation in delivery of urologic education.
2. Greater monitored operative supervision for urological trainees.
3. Laying down of annual targeted training objectives.
4. Development of an objective manual of assessing competencies and regular appraisal.
5. Encouraging trainees towards subspecialization appropriate to service demand. Creating ‘Jack of all (most), king of one’ urologists.
6. Intensive short ‘Hands on’ laboratory curriculum for residents for improving skills in laparoscopy and endourology.[13,14]

These include some of the suggestions which could possibly enhance the utility of our current training program. Establishment of a Steering Committee to further delve into the requirements and mechanics of implementing appropriate evidence-based changes with a national consensus would be a logical step.

## INTRODUCTION OF FELLOWSHIPS AFTER BASIC UROLOGY RESIDENCY

“Fellowships enhance the overall quality of a training program and aid in faculty recruitment and retention. The future of academic urology is totally dependent on the “fellowship pipeline,” while in private practice (with the exception of pediatrics) it is not essential for most practice groups.”[10]

The introduction and enhancement of fellowship training in the various subspecialty areas is likely to improve growth of the subspecialties which will help retain the cutting edge of our specialty. This will also allow the PG program to develop a urologist with ‘core’ competency and not burden the program with extensive in-depth schedules. Yet, residents and fellows headed toward academic careers can be nurtured to develop skills in teaching, research and writing.

I envisage the use of ‘Fellowships’ to enhance the skill and depth of knowledge of the fellow and NOT be used as “another pair of hands” by the institute imparting the fellowship. Thus institutions entrusted with this role must be chosen well, on the basis of their merit, and their proven record of excelling in that subspecialty. The fellowships must be controlled by the Urological Society of India (USI), under the aegis of the Medical Council of India (MCI), rather than a particular university. This will allow institutions, not necessarily under any particular university, to also be included for such programs. These centers should have a regular appraisal of the program to ensure maintenance of the requisite standards and continuation of the program at that center.

Continuing medical education (CME) programs and introduction of ‘mini-fellowships’[15] for practicing urologists can further improve upon the pool of ‘Jack of all, king of one’ urologists, as well as update the practice of urology.

## CONCLUSION

The current PG program in its three-year format still provides the necessary training required to help shape a ‘core’ urological workforce suited to the service requirement of the country. Enhancement of the ‘art’ of urology in the country must rely upon training in the various well-recognized subspecialties, which should be imparted as a fellowship in an objective and focused manner. Flexibility and tailoring (to match the skills and desires of the individual trainee) are important ingredients of any training system that hopes to respond to a rapidly changing environment.

## References

[1] List of Registered urologists- USI. http://www.usi.org.

[2] (2001). Estimated population in India in July 2007. Census of India.

[3] The Council of the British Association of Urological Surgeons (2002). The Provision of Urological Services in the UK.

[4] 51st AIIMS Annual Report: 2006-2007. Department of Urology statistics.

[5] (2007). St. Stephen's Hospital, Urology OT register.

[6] (2007). Sir Ganga Ram Hospital, Urology OT records.

[7] Payne SR, Shaw MB (2005). What impact will shortened training have on urological service delivery?. Ann R Coll Surg Engl.

[8] Pearce I, Royle J, O'Flynn K, Payne S (2003). The record of in-training assessments (RITAs) in urology: An evaluation of trainee perceptions. Ann R Coll Surg Engl.

[9] Klotz LH (2003). What residents need. Can J Urol.

[10] Future of urologic residency strategic planning committee The future of urology and urologic education in America. AUA News.

[11] Steers WD, Schaeffer AJ (2005). Is it time to change the training of urology residents in the United States?. J Urol.

[12] Carroll PR (2006). Modifying urology residency training: Time to speed up or slow down?. J Urol.

[13] Traxer O, Gettman MT, Napper CA, Scott DJ, Jones DB, Roehrborn CG (2001). The impact of intense laparoscopic skills training on the operative performance of urology residents. J Urol.

[14] Chatterjee S, Radomski SB, Matsumoto ED (2007). Durability of endourologic skills: Two-year follow-up study. J Endourol.

[15] McDougall EM, Corica FA, Chou DS, Abdelshehid CS, Uribe CA, Stoliar G (2006). Short-term impact of a robot-assisted laparoscopic prostatectomy ‘mini-residency’ experience on postgraduate urologists' practice patterns. Int J Med Robot.

